# OZITX, a pertussis toxin-like protein for occluding inhibitory G protein signalling including Gα_z_

**DOI:** 10.1038/s42003-022-03191-5

**Published:** 2022-03-23

**Authors:** Alastair C. Keen, Maria Hauge Pedersen, Laura Lemel, Daniel J. Scott, Meritxell Canals, Dene R. Littler, Travis Beddoe, Yuki Ono, Lei Shi, Asuka Inoue, Jonathan A. Javitch, J. Robert Lane

**Affiliations:** 1 Drug Discovery Biology, Monash Institute of Pharmaceutical Sciences, Monash University, Parkville, VIC 3052 Australia; 2 Division of Physiology, Pharmacology and Neuroscience, School of Life Sciences, Queen’s Medical Centre, University of Nottingham, Nottingham, UK; 3 Centre of Membrane Proteins and Receptors, University of Birmingham and University of Nottingham, Nottingham, UK; 4 Departments of Psychiatry and Molecular Pharmacology and Therapeutics, Vagelos College of Physicians and Surgeons, Columbia University, New York, NY USA; 5 Division of Molecular Therapeutics, New York State Psychiatric Institute, New York, NY USA; 6 NNF Center for Basic Metabolic Research, Section for Metabolic Receptology, Faculty of Health and Medical Sciences, University of Copenhagen, Copenhagen, Denmark; 7 Department of Biochemistry and Molecular Biology, University of Melbourne, Parkville, VIC 3052 Australia; 8 The Florey Institute of Neuroscience and Mental Health, University of Melbourne, Parkville, 3052, VIC 3052 Australia; 9 Infection and Immunity Program and Department of Biochemistry and Molecular Biology, Biomedicine Discovery Institute, Monash University, Clayton, VIC 3052 Australia; 10 Department of Animal, Plant and Soil Science and Centre for AgriBioscience, La Trobe University, Bundoora, VIC 3086 Australia; 11 Graduate School of Pharmaceutical Sciences, Tohoku University, Sendai, Miyagi 980-8578 Japan; 12 Computational Chemistry and Molecular Biophysics Section, National Institute on Drug Abuse - Intramural Research Program, National Institutes of Health, Baltimore, MD USA

**Keywords:** Receptor pharmacology, Cell signalling

## Abstract

Heterotrimeric G proteins are the main signalling effectors for G protein-coupled receptors. Understanding the distinct functions of different G proteins is key to understanding how their signalling modulates physiological responses. Pertussis toxin, a bacterial AB_5_ toxin, inhibits Gα_i/o_ G proteins and has proven useful for interrogating inhibitory G protein signalling. Pertussis toxin, however, does not inhibit one member of the inhibitory G protein family, Gα_z_. The role of Gα_z_ signalling has been neglected largely due to a lack of inhibitors. Recently, the identification of another Pertussis-like AB_5_ toxin was described. Here we show that this toxin, that we call OZITX, specifically inhibits Gα_i/o_ and Gα_z_ G proteins and that expression of the catalytic S1 subunit is sufficient for this inhibition. We identify mutations that render Gα subunits insensitive to the toxin that, in combination with the toxin, can be used to interrogate the signalling of each inhibitory Gα G protein.

## Abstract (web summary)

A recently identified pertussis toxin-like AB5 toxin, OZITX, is found to inhibit Gαi/o and Gαz G proteins. In combination with directed mutations, it is a useful tool for interrogating Gαi/o/z G protein subunits individually.

## Introduction

Heterotrimeric guanine nucleotide-binding proteins (G proteins) are important signalling transducers that link cell-surface receptors such as G protein-coupled receptors (GPCRs) to intracellular effectors^1–3^. They consist of a Gα subunit as well as Gβ and Gγ subunits that function as an obligate dimer. There are four subfamilies of Gα subunits (Gα_s_, Gα_i_, Gα_q_ and Gα_12_) based on sequence similarity. Their functions can be broadly generalised based on this classification. The stimulatory (Gα_s_) and the inhibitory (Gα_i_) subfamilies stimulate and inhibit adenylate cyclases, respectively^1,4^. The Gα_q_ subfamily activates phospholipase C-β leading to increases in cytosolic Ca^2+^, and the Gα_12_ subfamily activates Rho family GTPases that regulate cytoskeletal processes^2,5^. Understanding the distinct signalling roles of individual members of each subfamily is central to our comprehension of how they control different physiological processes.

The Gα subunit, and in particular its carboxy tail, is largely responsible for determining the specificity of the interaction with an activated GPCR^6,7^. The GPCR acts as a guanine nucleotide exchange factor, promoting the exchange of bound GDP for GTP at the guanine nucleotide-binding domain of the Gα subunit. This causes the Gα subunit to dissociate from, or rearrange relative to, the Gβγ dimer, and both then act on downstream effectors^8,9^. The Gα subunit is a GTPase, hydrolysing GTP to restore GDP to the binding domain, allowing the Gβγ dimer to reassociate and completing the cycle.

AB_5_-type toxins have proved to be useful tools for the interrogation of G protein signalling. These toxins are characterised by a hetero-hexameric structure consisting of the enzymatically active A subunit and pentameric ring of B subunits, which are responsible for recognition of host cell-surface receptors and facilitate cellular entry. In order to modulate host cell behaviour, AB_5_ toxins have varied actions on their targets, including protease activity^10^, RNA N-glycosidase activity^11^ and ADP-ribosylation^12^. Of relevance to G-protein signalling, cholera toxin acts on the Gα_s_ subfamily^13^ and Pasteurella multocida *Pasteurella multocida* toxin acts on the Gα_i_, Gα_q_ and Gα_12_ family to render them constitutively active^14^. Pertussis toxin (PTX), from *Bordetella pertussis*, ADP ribosylates all members of the Gα_i_ subfamily, except for Gα_z_^15^. Researchers have exploited these actions to identify the Gα subunits responsible for particular cell signalling processes. PTX-driven ADP ribosylation occurs on a cysteine residue of four residues from the carboxy terminus of Gα_i_ subunits, rendering them incapable of coupling to GPCRs. PTX-insensitive Gα_i/o_ subunit mutants, in which the cysteine modified by PTX is replaced by another residue, have been used to understand the role of individual Gα_i_-subfamily members in vitro. One inhibitory G-protein family member, Gα_z_, lacks this cysteine and is thus insensitive to PTX. Gα_z_ has a slow GDP-GTP exchange rate, slow GTP hydrolysis rate, and a restricted pattern of expression^16–19^. Despite these unique characteristics, relatively little is known about the physiological role of Gα_z_ signalling, although evidence has been provided for its roles in circadian behaviours, dopaminergic signalling, and pancreatic islet β cell biology^19–22^. Its function in other physiological processes remains elusive, in part due to its insensitivity to PTX. Indeed, there may be cases in which inhibitory G protein signalling has been ruled out based on a lack of PTX effect while neglecting the potential role of Gα_z_.

A recent publication reported the identification and structural characterisation of a PTX-like protein derived from a uropathogenic *Escherichia coli*^23^. The toxin has an active A subunit homologous to that of PTX and has a similar overall structural fold (Supplementary Fig. 1). Application of this toxin to HEK 293 cells, African green monkey kidney cells or bovine brain lysate revealed its substrates as heterotrimeric G proteins^23^. Using Gα_i2_ as a substrate in vitro, the toxin was shown to have distinct site(s) of ADP ribosylation from that of PTX—an asparagine residue and a lysine residue eight and ten residues from the carboxy terminus, respectively^23^. The asparagine is conserved across several Gα subunits, suggesting that the toxin may have broader substrate specificity than PTX.

In this study, we show that this toxin inhibits the coupling of all Gα_i/o/z_ G proteins, including Gα_z_. Thus, we refer to it as Gα_O_, Gα_Z_ and Gα_i_ inhibiting ToXin, or in short; OZITX. The active A subunit is functional when expressed in mammalian cells, bypassing the need for toxin purification. Moreover, we generate mutants of the members of the Gα_i_ subfamily that are OZITX insensitive, and hence, can serve as tools in combination with OZTIX treatment to investigate the role of individual Gα_i/o/z_ G proteins.

## Results

### OZITX treatment abolishes GPCR-mediated activation of all Gα_i_ subfamily members, including Gα_z_

**Fig. 1:**
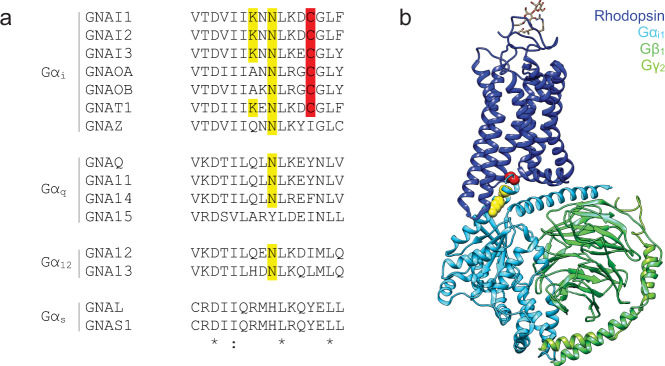
Identification of Gα carboxy-tail amino acid residues that are putatively ADP-ribosylated by OZITX. **a** Amino acid sequence alignment of carboxy-termini residues of heterotrimeric Gα proteins. Sequences were aligned with Clustal Omega version 1.2.4. ‘*’ represents a completely conserved residue. ‘:’ represents a conserved residue (>0.5 in the Gonnet PAM 250 matrix). ‘.’ represents a weakly conserved residue (≤0.5 and >0 in the Gonnet PAM 250 matrix). Cysteine residues ADP ribosylated by PTX are indicated in red. Putative lysine and asparagine residues ADP ribosylated by OZITX identified by Littler and colleagues^23^ are indicated in yellow. The asparagine residue that is a putative substrate is conserved across many Gα subunits. **b** The location of OZTX’s and PTX’s substrate amino acid sites within a GPCR–G protein complex. The structure of rhodopsin bound to Gα_i1_β_1_γ_2_ is depicted in the cartoon (PDB code 6CMO). Rhodopsin is shown in dark blue, Gα_i1_ in light blue, Gβ_1_ in green and Gγ_2_ in light green. The carboxy-terminal Cys^351^ residue ADP ribosylated by PTX is shown in red spheres. Lys^345^ and Asn^347^, the putative residues ADP ribosylated by OZITX, are highlighted in yellow spheres. Graphic constructed using UCSF chimera.

**Fig. 2:**
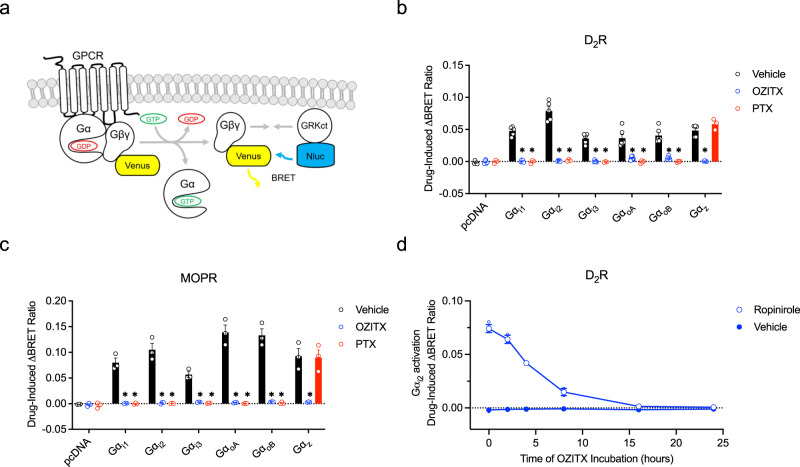
Activation of members of the Gαi subfamily in the presence of OZITX and PTX. **a** Representation of the BRET sensors used for detection of G-protein activation. The Gαβγ heterotrimer is activated through agonist binding to the GPCR and the Gα and Gβγ-venus dissociate. Free Gβγ-venus is bound by masGRK3ct-Nluc that serves as a BRET donor resulting in non-radiative energy transfer from Nluc to venus. **b** Dopamine D_2_receptor (D_2_R)-mediated activation or (**c**) μ opioid receptor (MOPR)-mediated activation of Gα_i_ subfamily members in the presence of OZITX or PTX. HEK293A CRISPR/Cas ΔGα-all cells expressing the D_2_R or MOPR were pre-treated with either vehicle (black), OZITX (blue) or PTX (red) for 24 h. Cells were stimulated with 1 μM ropinirole (D_2_R) or 1 μM DAMGO (MOPR) for 2.5 min followed by BRET detection. Data represent the mean drug-induced increase in BRET ratio from vehicle ± SEM from six independent experiments (D_2_R) or three independent experiments (MOPR). *Represents where the response is significantly different (*P* < 0.001) from the respective vehicle toxin untreated control condition (black bar) as determined by a one-way ANOVA with Dunnett’s multiple comparisons test. **d** Time course of OZITX treatment on G-protein activation. HEK 293 ΔG**α**-all cells were transfected with cDNA encoding the D_2L_R, Gα_i2_ and G-protein activation sensors. Cells were pre-treated with OZITX for the indicated times. BRET was measured 2.5 min after stimulation with 1 µM ropinirole (blue open circles) or vehicle (blue filled circles). The basal BRET ratio prior to agonist stimulation has been subtracted to give the drug-induced ΔBRET ratio. Data represent the mean ± SD from three separate experiments. Individual replicates are shown as small circles.

We hypothesised that OZITX may display a broader selectivity as compared to PTX because the Asn^348^ residue that is ADP ribosylated by OZITX is conserved in a greater number of Gα subunits as opposed to the cysteine modified by PTX, which is only present in Gα_i_ and Gα_o_ family members (Fig. 1a, b)^23^. We first sought to determine whether OZITX inhibits coupling to members of the inhibitory G protein subfamily. To achieve this, we used a previously described bioluminescence resonance energy transfer (BRET) assay that measures the release of Gβγ subunits from the Gα subunits upon activation of the heterotrimer (Fig. 2a)^24,25^. While this assay provides a method for rapidly assessing G protein activation, the signal may be partially contaminated by endogenously expressed Gα subunits^25,26^. We, therefore, adapted the assay for use in HEK293A CRISPR/Cas ΔGα-all cells in which all the Gα subunits had been genetically knocked out^27^. This allowed us to monitor the Gβγ release specifically from the activation of one Gα subtype of interest that had been exogenously transfected.

The dopamine D_2_ receptor (D_2_R) promiscuously couples to Gα_i/o_ and Gα_z_ G proteins^28,29^. Cells transiently expressing the D_2_R were pre-incubated with PTX or OZITX followed by stimulation with the D_2_-like receptor-selective agonist ropinirole^30^. We observed that OZITX completely blocked the activation of Gα_i1_, Gα_i2_, Gα_i3_, Gα_oA_ and Gα_oB_ (Fig. 2b). Further, as predicted from the carboxy-tail Asn^348^ residue presented in Gα_z_, Gα_z_ could no longer couple to the D_2_R following OZITX treatment as well (Fig. 2b). In contrast, Gα_z_ was insensitive to inhibition by pre-treatment of cells with PTX, consistent with the absence of the required cysteine residue (Fig. 1a). This finding extends the initial characterisation of OZITX, showing that unlike PTX it can inhibit Gα_z_ as well as Gα_i/o_^23^.

Next, analogous experiments were performed with another Gα_i/o/z_-coupled GPCR; the μ opioid receptor. HEK293A CRISPR/Cas ΔGα cells transiently expressing the MOPR were pre-incubated with either OZITX or PTX and then stimulated with the agonist DAMGO (Fig. 2c). OZITX inhibited coupling to each of the Gα_i/o/z_ subunits completely (Fig. 2c). This showed, as expected, that OZITX does not discriminate between GPCRs when inhibiting Gα_i/o/z_ G-protein activation.

We then sought to further characterise the toxin by measuring the activation of Gα_i2_ by the D_2_R after exposure to OZITX at different timepoints. Gα_i2_ activation decreased with increasing time of OZITX exposure until Gα_i2_ activation was completely abolished approximately sixteen hours after the addition of OZITX (Fig. 2d). This is consistent with the characteristics of PTX and suggests that OZITX, like PTX, would be best utilised in the laboratory by incubating with the cells for more than 16 h (Supplementary Fig. 2).

### OZITX does not ablate Gα_s_, Gα_q_ or Gα_12_ subfamily coupling

**Fig. 3:**
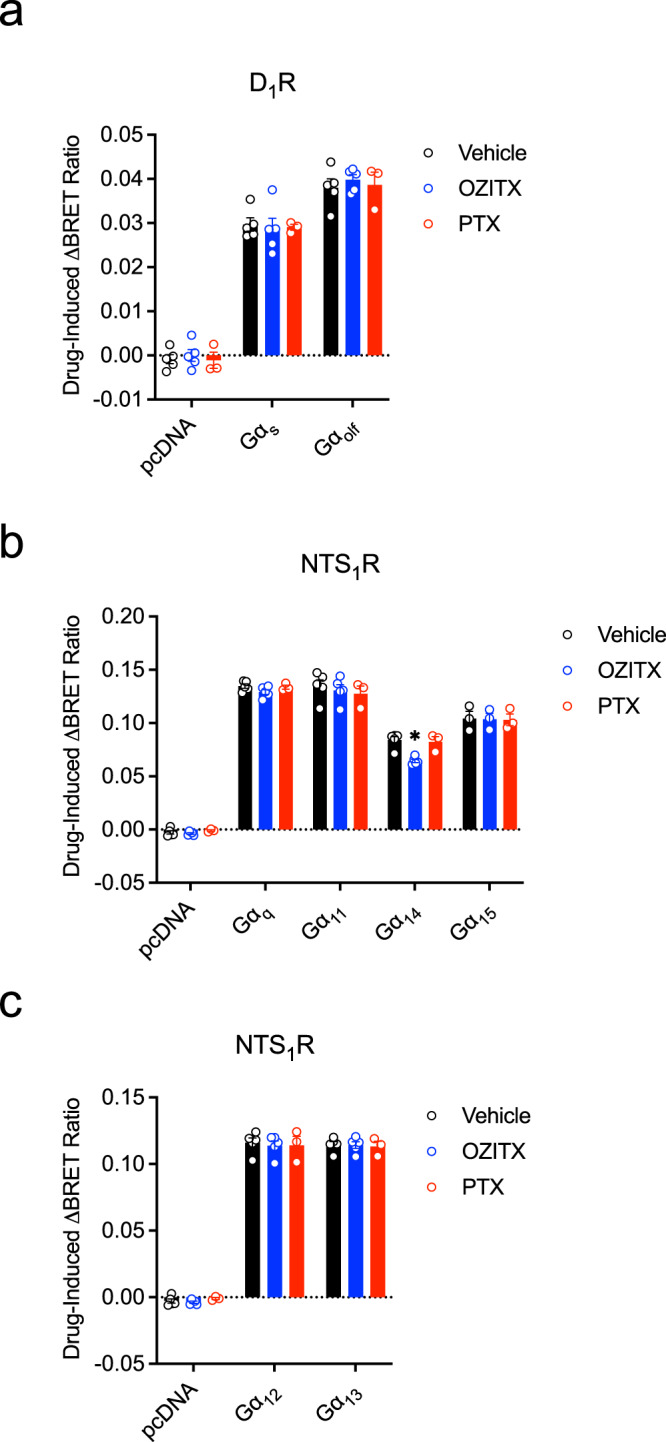
Gαs, Gαq and Gα12 subfamily activation in presence of OZITX and PTX. **a** Activation of Gα_s_ subfamily members by the dopamine D_1_ receptor (D_1_R) in the presence of OZITX and PTX. **b** Activation of Gα_q_ subfamily members by NTS_1_R in the presence of OZITX and PTX. **c** Activation of Gα_12_ subfamily members by the neurotensin receptor 1 (NTS_1_R) in the presence of OZITX and PTX. HEK 293 ΔGα-all CRISPR cells were transfected with cDNA encoding the particular GPCR and Gα together with the G-protein activation sensors as described in “Methods”. The cells were pre-treated with either vehicle (black), OZITX (blue) or PTX (red) for 24 h before stimulation with the agonists 100 nM SKF83822 (D_1_R)/1 μM NT8-13 (NTS_1_R) for 2.5 min followed by BRET detection. The data are represented as the mean drug-induced increase in BRET ratio from vehicle control ± SEM from three separate experiments. *Represents where the OZITX or PTX-treated condition is significantly different (*P* < 0.001) from the vehicle-treated condition (black) as determined by a one-way ANOVA with Dunnett’s multiple comparisons test.

In addition to the inhibitory Gα G protein subfamily, the asparagine eight residues from the carboxy terminus is conserved in other Gα subfamily members (Fig. 1a). We therefore sought to further assess the substrate selectivity of OZITX across all Gα subunits. The Gα_s_ subfamily possesses a histidine residue instead of an asparagine in this position. In accordance with OZITX’s proposed mechanism of action, overnight incubation with OZITX did not inhibit Gα_s_ or Gα_olf_ activation by the dopamine D_1_ receptor, a Gα_s/olf_-coupled receptor, stimulated with the D_1_R-selective agonist SKF83822 (Fig. 3a)^31–34^.

Gα_q_, Gα_11_ and Gα_14_, but not Gα_15_, possess an asparagine residue eight residues from their C termini, so one might expect these three subunits to be substrates for OZITX (Fig. 1a). We measured the activation of the Gα_q_ subfamily proteins by the Gα_q_-coupled neurotensin receptor 1 stimulated by the agonist NT8-13 with and without OZITX treatment^35,36^. OZITX pre-treatment did not inhibit the activation of Gα_q_, Gα_11_ or Gα_15_ although Gα_14_ activation was slightly (~25%) decreased (vehicle control = 0.0840, OZITX-treated = 0.0644, *P* = 0.0012, one-way ANOVA with Dunnett’s multiple comparisons test) (Fig. 3b).

Both members of the Gα_12_ subfamily; Gα_12_ and Gα_13_, also have asparagine as their eighth to last residue (Fig. 1a). The neurotensin receptor 1 is known to couple to the Gα_12_ subfamily^37^. While we were successful in detecting robust activation of Gα_12_ and Gα_13_, there was no inhibitory effect on the activation of either subunit when the cells were treated with OZITX (Fig. 3c). Taken together, we conclude that despite the presence of this asparagine residue at the C-terminus of Gα_q_ and Gα_12_ subfamily members, no detectable inhibitory action of OZITX was observed, with the exception of the limited inhibition of Gα_14_.

### Inhibition of cAMP production by Gα_i2_-, Gα_oA_- and Gα_z_ is inhibited by OZITX

**Fig. 4:**
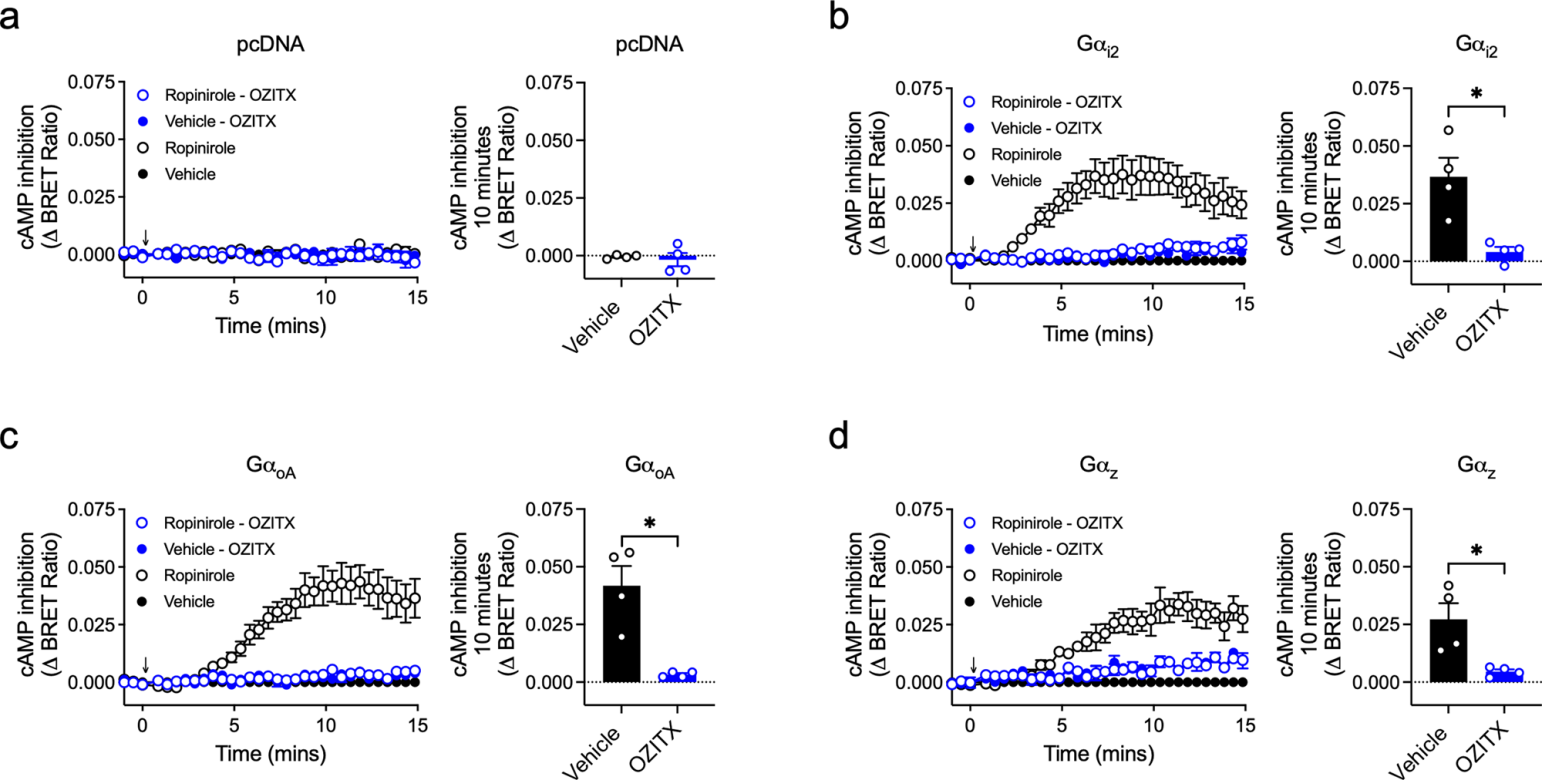
The effect of OZITX on Gαi2-, GαoA- and Gαz-mediated inhibition of cAMP production. Inhibition of forskolin-stimulated cAMP production was detected in live cells using CAMYEL; a conformational BRET sensor based on EPAC. HEK 293 ΔGα_i/o_ CRISPR cells were transfected with DNA encoding the D_2_R, CAMYEL and either (**a**) pcDNA3.1+ control, (**b**) Gα_i2_, (**c**) Gα_oA_ or (**d**) Gα_z_. Transfected cells were then incubated with either vehicle (black) or OZITX (blue) for 24 h. Cells were then pre-stimulated with 10 µM forskolin for 10 min before stimulation with either vehicle control (filled circles) or 1 μM ropinirole (open circles). Data are baseline-corrected to the cells not treated with OZITX or ropinirole and is shown as the mean ± SEM from four separate experiments. Measurements of cAMP inhibition between vehicle and OZITX-treated conditions were compared using an unpaired Student’s *t* test * represents statistical significance *P* < 0.05 (pcDNA –*P*= 0.700, Gα_i2_ −*P* = 0.008, Gα_oA_ – *P* = 0.004_,_ Gα_z_ – 0.019).

Cell-surface receptor signalling is commonly amplified in subsequent steps down the signalling cascade. We wanted to confirm that the apparent complete blockade of Gα_i/o/z_ signalling at the level of G-protein coupling would concord with measurements further downstream. We assessed the effect of OZITX treatment in measurements of intracellular cAMP levels using an intramolecular conformational BRET sensor of cAMP (CAMYEL), since the Gα_i_ subfamily bind and inhibit adenylate cyclases^4,38^. In these experiments, we used HEK293A cells that harbour a genetic knockout of all the Gα_i_ subfamily members using CRISPR/Cas (HEK293A CRISPR/Cas ΔGα_i_)^27^. Individual Gα_i_ subunits of interest were then transfected into this cell background. Cells were treated with forskolin to stimulate adenylate cyclase, followed by treatment with ropinirole to stimulate the D_2_R, leading to activation of the Gα_i/o/z_ subunit of interest. In the absence of a transfected Gα subunit, there was no detectable drug-induced inhibition of cAMP production (Fig. 4a). When Gα_i2_ or Gα_oA_ were transfected, activation of the D_2_R produced a decrease in relative cAMP levels (indicated by an increase in BRET ratio) and this was completely abolished in cells treated with OZITX (Fig. 4b, c). Cells transfected with Gα_z_ also produced a decrease in cAMP, albeit to a slightly smaller degree, and this was again blocked in the presence of OZITX (Fig. 4d). This confirms that OZITX-mediated ADP ribosylation inhibits downstream Gα_i/o/z_-mediated signalling.

### The active A subunit of OZITX can be transfected into mammalian cells to act as an inhibitor

**Fig. 5:**
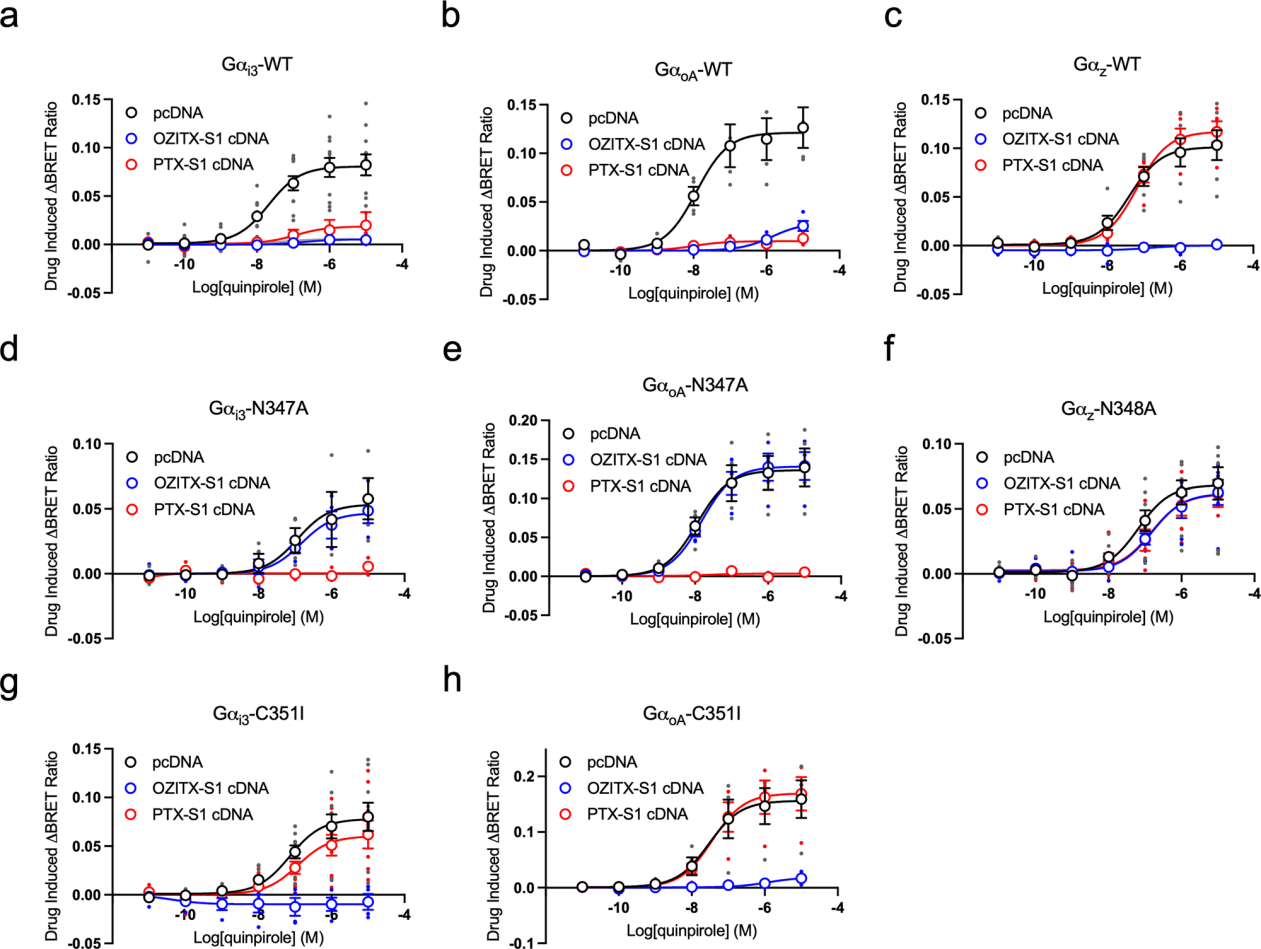
OZITX sensitivity of Gαi subfamily carboxy-tail Asn347/348 mutants. **a** Gα_i3_-WT activation, *n* = 11. **b** Gα_oA_-WT activation, *n* = 4. **c** Gα_z_-WT activation, *n* = 5. **d** Gα_i3_-N347A activation, *n* = 4. **e** Gα_oA_-N347A activation, *n* = 4. **f** Gα_z_-N347A activation, *n* = 6. **g** Gα_i3_-C351 activation, *n* = 4. **h** Gα_oA_-C351 activation, *n* = 4. The G-protein activation assay was performed on WT, Asn347Ala/Asn348Ala (putative OZITX site) and Cys351Ile (PTX-insensitive) mutants. Cells were transfected with the D_2_R, the particular Gα mutant, the G-protein activation sensors and either a pcDNA3.1+ control (black open circles), OZITX-S1 cDNA (blue open circles) or PTX-S1 cDNA (red open circles). Cells were then stimulated with increasing concentrations of quinpirole before BRET detection. Data represent the mean drug-induced increase in BRET ratio from vehicle ± SEM. Individual replicates are shown as small circles.

In order to treat cells with AB_5_ toxin protein complexes, both expression and purification of this toxin are required^23^. The active A subunit of PTX alone can be transiently expressed to inhibit Gα_i/o_ signalling^39,40^. Accordingly, we tested whether the OZITX would be functional upon transfection of the cDNA encoding the active A subunit alone (OZITX-S1), thus increasing its accessibility and utility to laboratories. The cDNA sequence of OZITX-S1 was codon-optimised for high expression in human cells and co-transfected into HEK293T cells along with the D_2_R, the WT Gα_i/o/z_ subunits and the G-protein activation sensors. Upon activation of the D_2_R with the agonist quinpirole the responses in cells transfected with Gα_i1-3_, Gα_oA_ and Gα_oB_ were inhibited in cells transfected with the positive control PTX-S1 cDNA as well as the OZITX-S1 cDNA (Fig. 5a–c and Supplementary Fig. 3). Importantly, while transfection of cells with OZITX-S1 inhibited Gα_z_ activation, transfection of PTX-S1 cDNA did not. Having shown that transfected OZITX-S1 is functional, we then confirmed the pattern of OZITX selectivity across the Gα subfamilies was in accord with our previous results using treatment with the complete OZITX protein complex (Supplementary Fig. 3). Consistent with these findings, the OZITX-S1 transfection was ineffective in abolishing the activation of Gα_s_, Gα_q_ and Gα_12_ subfamilies (Supplementary Fig. 3). In contrast to the partial inhibition of Gα_14_ that we observed when using the purified OZITX (Fig. 3b), we did not observe inhibition of Gα_14_ in experiments transiently expressing the OZITX-S1 (Supplementary Fig. 3).

ADP ribosylation of the C-terminal cysteine of Gα_i/o_ subunits by PTX is thought to inhibit the functional interaction between these G proteins and an activated GPCR. To test whether ADP ribosylation might also inhibit such an interaction we measured the recruitment of Gα_oA_:G_β_:G_γ_-venus G protein heterotrimers to the D_2L_R in the presence of either PTX-S1 or OZITX-S1 expression. Our data clearly show that both PTX and OZITX inhibit Gα_oA_:G_β_:G_γ_-venus G-protein heterotrimer recruitment to the D_2_R but that only OZITX inhibits Gα_z_:G_β_:G_γ_-venus G-protein heterotrimer recruitment, as expected. This is consistent with a mechanism of action whereby ADP ribosylation of the C-terminal asparagine of Gα_i/o/z_ by OZITX prevents their coupling to GPCRs akin to the action of PTX at the C-terminal cysteine of Gα_i/o_ (Supplementary Fig. 4).

### Gα_i_ subunits can be made OZITX insensitive for dissection of Gα_i/o/z_ subtype signalling specificity

Understanding the actions of a single Gα_i/o/z_ subtype can be challenging because there are usually multiple Gα_i/o/z_ members expressed within any given cell type. A method that has permitted the investigation of the role of individual Gα_i/o/z_ subunits in a particular signalling process, such as coupling to a specific GPCR, is the use of PTX-insensitive Gα_i_ mutants in combination with PTX to uncouple any endogenously expressed PTX-sensitive Gα_i_ subunits^41^. We, therefore, wanted to generate OZITX-insensitive Gα_i/o/z_ mutants in the hope of increasing the scope of OZITX applications. To render the Gα_i/o/z_ subunits insensitive to OZITX, we replaced the asparagine eight residues from the carboxy terminus to an alanine (Gα_i1_-N347A, Gα_i2_-N348A, Gα_i3_-N347A, Gα_oA_-N347A, Gα_oB_-N347A and Gα_z_-N348A) as this residue was previously identified as the most likely substrate site (Fig. 1a)^23^. We then performed G protein-activation assays using the D_2_R to activate each Gα_i_ mutant in the presence or absence of PTX-S1 or OZITX-S1 (Fig. 5 and Supplementary Figs. 5 and 6). In contrast to the activation of the wild-type Gα_i3_, Gα_oA_ and Gα_z_ that are all abolished by OZITX (Fig. 5a–c), activation of Gα_i3_-N347A, Gα_oA_-N347A and Gα_z_-N348A were OZITX insensitive (Fig. 5d–f). When these mutant Gα subunits were transfected the potency of quinpirole was similar to that observed in the case of the WT Gα subunit, suggesting that these mutations did not affect the efficiency with which they couple to the D_2_R. In addition, it was observed that the N347A/N348A mutation did not impact the PTX sensitivity of the Gα_i_ subunits (Fig. 5d–f). Likewise, the well-characterised PTX-insensitive mutation (C351I) introduced into Gα_i3_ and Gα_oA_, did not disturb the ability of OZITX to act on them (Fig. 5g, h). Having identified that the N347A/N348A mutation renders these Gα_i_ members insensitive to OZITX without perturbation, the mutations were extended into the remaining Gα_i_ subunits and validated (Supplementary Figs. 5 and 6).

### The C-terminal ten residues of Gα_i_ are sufficient to confer OZITX selectivity

The asparagine residue important for the inhibitory action of OZITX is present in both the G_i/o/z_ and the G_q_ subfamilies (with the exception of Gα_15_). Thus, the selective action of OZITX at the G_i/o/z_ G-protein α subunits appears not to be determined solely by the presence or absence of this residue. Similarly, a previous study has shown that replacement of the 5 C-terminal residues of Gα_q_ with that of Gα_i_ (which includes the cysteine that is modified by PTX) allows this chimeric Gα_qi5_ G protein to couple to G_i_ protein family-coupled receptors but, importantly, does not confer sensitivity to PTX^42^. This suggests that there are determinants of PTX in addition to the presence of this cysteine residue. To explore other determinants of OZITX selectivity we generated two chimeric G proteins in which the last 10 (Gα_qi10_) or 13 (Gα_qi13_) residues of Gα_q_ were replaced with those of Gα_i3_, a region that includes the asparagine residue modified by OZITX. In an assay measuring Ca^2+^ mobilisation, OZITX was unable to inhibit the Ca^2+^ response of the muscarinic M_3_ acetylcholine receptor co-expressed with Gα_q_ when activated by the agonist carbachol (Supplementary Fig. 7). In this Ca^2+^ mobilisation assay we were unable to detect a measurable response to the agonist ropinirole when the D_2_R was co-expressed with Gα_q_, but we observed responses when the D_2_R was co-expressed with both chimeric G proteins, meaning the D_2_R can couple to both Gα_qi10_ and Gα_qi13_. Interestingly, both PTX and OZITX could inhibit these responses, indicating that the last 10 residues of Gα_i_ are sufficient to confer the sensitivity to both PTX and OZITX inhibition on a Gα_q_ background (Supplementary Fig. 7). While both PTX and OZITX partially inhibited the Gα_qi10_ Ca^2+^ signal, the expression of both toxins completely inhibited Gα_qi13_ signalling. This pattern is consistent with the idea that the greater the amount of C-terminal Gα_i_ amino acids swapped with those of Gα_q_, the better the chimeric Gα_qi_ proteins become as substrates for both αβ_5_ toxins. However, the lower potency of ropinirole in the Gα_qi13_ Ca^2+^ assay suggests that the coupling of the D_2_R to this chimera is less efficient, which may also account for the apparently greater inhibitory effect of PTX and OZITX (Supplementary Fig. 7).

## Discussion

For decades, PTX and CTX have proven to be useful tools in GPCR signalling research to interrogate the Gα protein subfamilies or even specific Gα proteins responsible for particular physiological processes. Here, we characterise and demonstrate the utility of OZITX, a recently identified AB_5_ toxin, for the inhibition of GPCR-mediated activation of the Gα_i/o/z_ subfamily. Importantly, unlike PTX, this activity extends to include Gα_z_. OZITX acts to ADP-ribosylate an asparagine in the C-terminus of Gα_i/o/z_ proteins, a site distinct from the cysteine modified by PTX, accounting for this broader specificity. We found that OZITX displays a selective action to completely inhibit Gα_i/o/z_ proteins with no activity at Gα_s_, Gα_q_ or Gα_12_ proteins, with the exception of limited inhibition of Gα_14_. The catalytic subunit of PTX (PTX-S1) can be expressed in mammalian cells to effectively inhibit Gα_i/o_ signalling, avoiding the time and cost associated with acquiring the purified protein^39,40^. We demonstrate that the catalytic OZITX-S1 subunit can be used in a similar manner, increasing the utility of this tool. We identify mutations within Gα_i/o/z_ subfamily members that render them insensitive to OZITX and maintain their ability to couple to GPCRs. Together, these tools can be used to identify the Gα_i/o/z_ subunits participating in defined signalling pathways.

PTX played an important role in identifying the Gα_i_ subfamily by distinguishing it from the Gα_s_ subfamily^4^. PTX was shown to block the inhibitory effect that Gα_i_ proteins have on adenylyl cyclases, thus building evidence for a separate Gα species with distinct functionality to Gα_s_. Since then, PTX has been widely used with the same rationale, that is, to differentiate GPCR responses mediated by Gα_i_ proteins from other signal transducers^43^. However, such an approach cannot exclude the possibility that Gα_z_ might contribute to a particular response since it is insensitive to PTX^17,18^. A clear advantage of OZITX, then, is that it can inhibit Gα_z_ in addition to inhibiting Gα_i1_, Gα_i2_, Gα_i3_ and the Gα_o_ isoforms. We have not evaluated whether OZITX inhibits the coupling of the visual and taste Gα subunits; Gα_t1_, Gα_t2_ and Gα_gust_. One might expect ADP ribosylation by OZITX to occur on Gα_t1_, Gα_t2_ and Gα_gust_ since they harbour an asparagine as their eighth to last amino acid residue in addition to having high sequence homology to the other Gα_i_ subunits, although given our findings that not all Gα subunits that contain this asparagine are inhibited by OZITX, inhibitor activity at Gα_t1_, Gα_t2_ and Gα_gust_ must be determined experimentally.

Our findings suggest that OZITX could serve as a replacement for PTX in most experimental paradigms aimed at interrogating Gα_i/o/z_ G-protein signalling. There are, however, cases where PTX and OZITX can be used in parallel due to their different Gα specificities, for example when disentangling the functions of Gα_z_ from the other Gα_i/o_ subunits. OZITX-treated, PTX-treated and -untreated conditions run in parallel would enable the signalling mediated by Gα_z_, PTX-sensitive Gα_i_ subunits and toxin-insensitive Gα subunits to be isolated.

Previous studies aimed at interrogating Gα_z_ signalling have relied on other strategies, including overexpression of Gα_z_-specific RGS proteins^19^, Gα_z_-directed siRNA^44^, and Gα_z_ de-activation via PKC phosphorylation^45^. However, unlike OZITX, these approaches do not completely block activation of Gα_z_ so the influence of residual Gα_z_ signalling cannot be excluded, particularly when looking at an effect further down an amplified signalling cascade. Genetic knockouts of the gene that encodes Gα_z_ have been used for this reason but are technically challenging as compared to OZITX treatment^20,46^. In addition, the results of such knockout approaches may be confounded by adaptive changes to the cell and/or circuit over time that compensate for the loss of that particular protein. The advantage of OZITX is that it can be used in a relatively acute manner following overnight treatment, so its use is less likely to be confounded by compensatory changes in cell function.

The substrate site that is ADP ribosylated by OZITX was shown to be an asparagine eight residues from the C-terminus of the Gα subunit^23^. In agreement with this, we showed that Gα_i_ subunits can be made OZITX insensitive through mutation of the aligned asparagine in this position. Within our set of experiments, we observed that these mutations did not affect the potency or magnitude of the measured response as compared to when the WT Gα was used. This indicates that this mutation has not changed the coupling efficiency between the receptor and Gα subunit. It should be acknowledged, however, that these observations may be both receptor and downstream effector dependent. It may be that other mutations at this position may be superior to the alanine mutation for a particular combination, as has been observed for analogous studies using PTX and PTX-insensitive Gα mutants^47^. Nonetheless, the OZITX-insensitive mutants can serve as a useful tool in combination with OZITX treatment to investigate the signalling of particular Gα_i/o/z_ proteins in isolation. In our hands, mutation of the Asn^347/348^ residue alone was sufficient to render Gα_i1_, Gα_i2_ and Gα_i3_ resistant to OZITX. These Gα subunits contain a lysine residue as their tenth-to-last residue (Lys^345/346^) that was suggested to also be a site for OZITX-mediated ADP ribosylation by Littler and colleagues (Fig. 1a)^23^. Our results suggest that this Lys^345/346^ site is either a secondary substrate site that is minimally ADP ribosylated by OZITX, that ADP ribosylation of this site has no effect on G-protein coupling despite this residue being in close proximity to the GPCR upon coupling, or that the ribosylation of this lysine occurs sequentially to that of Asn^347/348^ (Fig. 1b), such that the mutation of the asparagine residue is sufficient to abrogate reaction.

We hypothesised that the presence of an asparagine residue eight residues from the C-terminus of various Gα subunits would confer sensitivity to OZITX in a similar manner to the way in which the presence of Cys^351/352^ confers sensitivity to PTX. In agreement with this, Gα_i/o/z_ proteins were inhibited by OZITX whereas Gα_s_, which lacks this asparagine, was not. We observed, however, that OZITX only had a small inhibitory effect on Gα_14_ activation and had no effect on the remaining Gα_q_ and Gα_12_ subunits despite the presence of the aligned asparagine. Thus, this asparagine is not the only determinant of selectivity. This lack of OZITX sensitivity can be reconciled either with OZITX not ADP-ribosylating the asparagine residue in G_q_ and G_12_ family Gα subunits or with ADP ribosylation of this residue in Gα_q_ and Gα_12_ not affecting GPCR coupling and signalling. Prior studies have shown that swapping the five carboxy-terminal residues of Gα_i2_ or Gα_oA_ onto Gα_q_ is not sufficient to confer sensitivity to PTX, even though the modified Gα_q_ contains the required cysteine residue four amino acids from the carboxy-termini^42^. This indicates that carrying the required substrate amino acid site is not sufficient to render the Gα subunit sensitive to PTX-like AB_5_ toxins. Our own experiments using chimeras in which the last ten or thirteen amino acids of Gα_q_ were swapped with those of Gα_i2_ revealed that exchange of this region was sufficient to confer both PTX and OZITX sensitivity. We computationally explored the feasibility of ADP-ribosylation of Gα subunits in the D_2_R-Gα_i_ and the 5HT_2A_–Gα_q_ complexes, by covalently docking the ADP-ribose moiety on the asparagine residue using the available cryo-EM structures of these complexes^48,49^. Our results reveal that the 5HT_2A_–Gα_q_ complex can easily accommodate the ADP-ribosylated asparagine whereas this covalent docking approach could not identify any feasible pose for the D_2_R–Gα_i_ complex without steric clash (Supplementary Fig. 8). Even though ADP-ribosylation of the asparagine can be sterically accommodated in the 5HT_2A_–Gα_q_ complex, we cannot rule out the possibility that this might still impact coupling, and as we have noted above, the residue may simply not be ADP-ribosylated due to the absence of other key determinants beyond the asparagine itself. Further studies are required to understand the additional structural basis for the recognition of specific Gα subunits by AB_5_-type toxins such as OZITX and PTX and to understand the selective action of OZITx for G_i/o/z_ family G proteins.

Our study illustrates the continuing value in the characterisation and use of AB_5_ toxins as laboratory tools. Host–pathogen interactions are hotspots of molecular evolution that result in proteins with extraordinary functionality. This is exemplified in the diversity of actions of ADP-ribosylating AB_5_ toxins including PTX and CTX and now OZITX and their resulting value as research tools.

## Methods

### Materials

Polyethylenimine (PEI), Linear (MW 25,000) was purchased from Polysciences, Inc. Ropinirole was purchased from BetaPharma (Shanghai) Co. Ltd. DAMGO ((_D_-Ala^2^, N-Me-Phe^4^, Gly-ol^5^)-enkephalin) was purchased from Mimotopes. SKF83822, neurotensin residues 8-13 (NT8-13), (−)-quinpirole hydrochloride (#1061), acetylcholine chloride (#A2661), carbachol (C4382), D-glucose (#G8270) and pertussis toxin (PTX) were purchased from Sigma-Aldrich. Isoproterenol (#1747) and endothelin-1 (#1160) were purchased from Tocris Bioscience (Bristol, UK). Coelenterazine-h was purchased from both NanoLight™ Technology and Dalton research molecules (#50303-86-9). Forskolin was purchased from Cayman Chemicals (#11018). Nano-Glo^®^ luciferase assay system, containing the furimazine substrate, was purchased from Promega.

### Plasmids

pcDNA3.1(+) encoding human constructs of long isoform of the dopamine D_2_ receptor (D_2L_R), μ opioid receptor (MOPR), dopamine D_1_ receptor (D_1_R), neurotensin receptor 1 (NTS_1_R), M_1_ muscarinic acetylcholine receptor (M_1_R), β_2_-adrenergic receptor (β_2_AR), endothelin A receptor (ET_A_R), Gα_i1_, Gα_i2_, Gα_i3_, Gα_oA_, Gα_oB_, Gα_z_, Gα_sS_, Gα_sL_, Gα_olf_, Gα_q_, Gα_11_, Gα_14_, Gα_15_-EE, Gα_12_ and Gα_13_ were from the cDNA Resource Centre (cDNA.org). pcDNA3L-His-CAMYEL was purchased from ATCC (ATCC MBA-277). masGRK3ct-Nluc, masGRK3ct-Rluc8, venus-1-155-Gγ_2_ and venus-156-239-Gβ_1_ were from Nevin Lambert, Augusta University. pCAGGS-Ric8A and pCAGGS-Ric8B were from Asuka Inoue, Tohoku University. The active S1 subunit of OZITX (*Ec*PltAB) was codon-optimised, synthesised and inserted into pcDNA3.1 + (see Supplementary Note 1 for sequence). OZITX-resistant mutations were made in Gα_i1_, Gα_i2,_ Gα_i3_, Gα_oA_ Gα_oB_ and Gα_z_ using site-directed mutagenesis. Primer sequences that were used for the mutagenesis can be found in Supplementary Table 1.

OZITX-resistant mutations were made by changing the eighth to last amino acid to an alanine (indicated in red) by using site-directed mutagenesis with the reverse primers used to the right, the alanine mutation change is shown in red and restriction sites chosen in blue (XhoI) or green (XbaI). The constructs were inserted into pcDNA3.1+ with KpnI and XhoI or XbaI as indicated. The two chimeric proteins Gα_qi10_ and Gα_qi13_ were generated using the Q5 site-directed mutagenesis kit from NEB. Primer sequences used for the mutagenesis can be found in Supplementary Table 2. PCR products were then treated with the KLD enzyme mix (kinase, ligase and DpnI) provided with the kit and then transform into NEB Turbo *E. coli* competent cells.

### Cell culture

HEK293T cells were purchased from ATCC (CRL-3216). HEK293A ΔGα-all CRISPR/Cas knockout cells and HEK293A ΔGα_i/o_ CRISPR/Cas knockout cells were generated as described before^27^. HEK293T cells, HEK293A ΔGα-all cells and HEK293A ΔGα_i/o_ cells were cultured in T175 flasks with Dulbecco’s Modified Eagle Medium (DMEM) + GlutaMAX^TM^-I (Gibco, Invitrogen, Paisley, UK) with 10% foetal bovine serum (Corning #35-010) and 1% penicillin/streptomycin (Corning #30-002). All Cells were grown in a humidified incubator in 5% CO_2_ at 37 °C and sub-cultured at a ratio of 1/10-1/20.

### Transfection

Briefly, cells were harvested from T175 flasks and plated into six-well Nunc™ tissue culture plates at a density of 500,000 cells per well. The following day, the media was removed and replaced with fresh media and transfected using PEI as the transfection reagent. The corresponding amounts of PEI and DNA were added to the buffer separately before mixing together, incubating for 20 minutes, and then adding dropwise on top of the cells in the fresh media.

For the G-protein-activation assays where the toxin was added exogenously: The HEK293A ΔGα-all CRISPR knockout cells were transfected using PEI in a ratio of 6:1 PEI:DNA (w/w) in PBS. The cells were transfected with a cDNA mixture consisting of: 0.143 µg GPCR, 0.286 µg Gα, 0.143 µg Gβ_1_-venus, 0.143 µg Gγ_2_-venus, 0.143 µg masGRK3ct-Nluc and 0.143 µg Ric8A or Ric8B or pcDNA3.1. The chaperone Ric8A was transfected together with Gα_14_ and Gα_15_ and Ric8B was transfected with Gα_olf_.

For the cAMP BRET assays, where the toxins were exogenously added: The HEK293A ΔGα_i/o_ CRISPR knockout cells were transfected using PEI in a ratio of 6:1 PEI:DNA (w/w) in PBS. The cells were transfected with a cDNA mixture consisting of: 0.143 µg D_2L_R, 0.286 µg Gα_i2_/Gα_oA_/Gα_z_/pcDNA3.1 and 0.429 µg CAMYEL sensor.

Assays, where the active A subunits of the toxins were transiently transfected, had the following conditions: HEK293T cells were transfected using PEI in a ratio of 1.5 PEI:1 DNA (w/w) mixed in 150 mM NaCl For the G-protein activation assays the cells were transfected with 0.500 µg β_1_, 0.500 µg Venus-γ_2_ and 0.100 µg masGRKctRluc8 as well as 1 µg of the G protein of interest together with 0.375 µg of a receptor suited for the specific G-protein class and 0.375 µg of the helper proteins Ric8A for Gα_14_ and Gα_15_ and Ric8B for Gα_olf_ and finally 0.200 µg of either the active subunit of PTX (PTX-S1), OZITX (OZITX-S1) or pcDNA3.1+ as a control. For the cAMP production inhibition assays the cells were co-transfected with 1 µg of the CAMYEL sensor (ATCC MBA-277). For the Ca^2+^ assay, the cells were transfected with 0.3 µg of the receptor (M3R or D2LR), 0.3 µg of Gα_q/qi10/qi13_ and 0.2 µg of either S1-PTX/S1-OZITX or pcDNA3.

### G-protein activation

G-protein activation was measured using a BRET assay that monitors Gβγ release^24,25^. The HEK293A ΔGα-all cells were first transfected as described earlier and the following day the cells were harvested and transferred into white 96-well CulturPlates (PerkinElmer) in DMEM + 10% FBS. In the conditions where the cells were treated with OZITX or PTX, the cells were left to adhere before being treated in the 96-well plate 16–20 h before performing the assay. The G-protein activation assays were then performed ~24 h after plating out the transfected cells. The media in each well was aspirated, washed with Hank’s balanced salt solution pH 7.4 (HBSS), replaced with HBSS and then kept at 37 °C for the remainder of the assay. Furimazine was added with a multi-stepper pipette 15 min before agonist addition and left to equilibrate. The agonist was then added, and cells were incubated in a LUMIstar Omega (BMG Labtech) plate reader. The BRET measurements were then taken 2.5 min after agonist addition. Simultaneous dual emission filters were used in the LUMIstar Omega for detection of the luciferase at 445–505 nm and venus at 505-565 nm, all measured at 37 °C. For G-protein activation assays where the toxin active A subunit cDNAs were transfected, the same protocol was followed with some exceptions: HEK293T cells were used instead of CRISPR/Cas gene-edited cells, DPBS + 5 mM glucose was used as the assay buffer, 96-well black-white isoplates were used, and the plate was detected five minutes after agonist stimulation in a PHERAstar FS (BMG Labtech). After acquiring the data, the ratio of the venus emission channel was then divided by the luciferase emission channel to determine the BRET ratio. The vehicle-subtracted raw BRET ratio (drug-induced increase in BRET) is plotted for the G-protein activation assay data.

### Gγ-mVenus recruitment assay

For the D_2_R-mediated Gγ-mVenus recruitment assay, HEK293T cells were seeded onto six-well plates and transfected with a 1:6 ratio of DNA:polyethylenimine with plasmids encoding D2R-nluc (50 ng), Gα_oA/z_ WT (cDNA resource centre, Bloomsburg University, PA, 125 ng), human Gβ1 (250 ng) and human Gγ2- mVenus (250 ng). Cells were harvested from six-well plates 24 h after transfection and plated into poly-D-lysine coated (Sigma-Aldrich) white-bottom 96-well optiplates (Wallac, PerkinElmer Life and Analytical Sciences) at a density of 50,000 cells per well. Twenty-four hours after cells were transferred to plates, media was aspirated, cells washed once with DPBS and 80 μL DPBS containing 5 mM glucose was added to each well. In all, 10 μL of coelenterazine was added to each well and the plate read on the PHERAstar FS (BMG Labtech) for 5 min, paused for the addition of 10 μL agonists and read again for 10 min. After acquiring the data, the ratio of the venus emission channel was then divided by the luciferase emission channel to determine the BRET ratio. The vehicle-subtracted raw BRET ratio (drug-induced increase in BRET) is plotted for the G-protein recruitment assay data.

### Gα_i/o/z_-mediated inhibition of cAMP production

The cAMP production inhibition assays’ principle is based on the ability of a genetically encoded conformational BRET sensor to detect the relative concentrations of intracellular cAMP^50^. Initially, the transfected HEK293A ΔGα_i/o_ cells were harvested and transferred into white 96-well CulturPlates in DMEM + 10% FBS 24 h after the transient transfection. When the cells were treated with OZITX or PTX, this occurred in the 96-well plate after adherence and about 18 h before the assay. Next, the cAMP inhibition assays were performed the following day after plating out the transfected cells and toxin or control treatment. On the day of the assay, the plate media was aspirated, washed once with HBSS pH 7.4 and replenished with HBSS pH 7.4 and then held at 37 °C for the rest of the experiment. In total, 5 μM coelenterazine-h was added 15 min before agonist addition. 10 µM Forskolin was added 10 min before agonist addition and the readings were then continuously taken in the live cells. Bioluminescence was detected on a LUMIstar Omega set to 37 °C. Simultaneous dual emission filters were used for the BRET donor at 445–505 nm and the acceptor at 505-565 nm. The ratio of the acceptor channel was then divided by the donor channel to determine the BRET ratio. The data was then baseline-corrected to the vehicle control wells over time. A slightly modified protocol was followed for the assays where the active subunit cDNAs of the toxins were transfected: HEK293T cells were used instead of the HEK293A ΔGα_i/o_ cells, 96-well black-white isoplates were used, DPBS + 5 mM glucose was used as the assay buffer, a higher concentration of 30 μM forskolin was used and this was co-added with the coelenterazine-h ten minutes prior to the addition of the agonist. The plate was then detected 20 min after agonist addition in a PHERAstar FS.

### Ca^2+^ mobilisation assays

Cells were seeded in a clear bottom black 96-well plate coated with poly-d-lysine (50 μg/ml) at 100,000 cells per well. The following day, cells were washed with 100 μl of 1× HBSS assay buffer supplemented with 10 mM glucose, 4 mM probenic acid at pH 7.4 and brilliant black, and then loaded with 100 μl Fluo-4 AM (1 μM) (prepared in DMSO and pluronic acid) for 45 min at 37 °C (no CO_2_). The release of Ca^2+^ was measured using a Flexstation 3 (Molecular Devices; Sunnyvale, CA). Drug dilutions were prepared in assay buffer (without Fluo-4) at 6x required concentration (volume 20 μl in 100 μl in Flexstation protocol) and transferred to a loading plate (transparent flat-bottom 96-well plate). Fluorescence was detected for 3 min30 s at 485 nm excitation and 525 nm emission. Relative fluorescence units were normalised to the fluorescence stimulated by ionomycin to account for differences in cell number and loading efficiency.

### Data analysis, statistics and reproducibility

GraphPad Prism 8 was used for data analysis and performing statistical tests. Statistical analysis was carried out with a Student’s *t* test or one-way ANOVA followed by a post hoc test where appropriate. *P* values <0.05 were considered statistically significant. Data sets were of at least *n* = 3 and the experimental *n* number is stated for each data set in the corresponding figure legend. Figures depicting molecular structures were constructed using ICM-Browser (MolSoft LLC) and UCSF Chimera^51^. The covalent docking was carried out with the covDock module of Schrodinger suite (version 2021-1), assuming a SN2 nucleophilic substitution reaction, which results in the a-orientation of the attached ADP-ribose moiety on the asparagine^52^.

### Reporting summary

Further information on research design is available in the Nature Research Reporting Summary linked to this article.

## Supplementary information

Peer Review File

Supplementary Information

Description of Additional Supplementary Files

Supplementary Data 1

Reporting Summary

## Data Availability

All original data have been deposited with *Communications Biology* in Supplementary Data 1 and are also available from the corresponding authors upon reasonable request.

## Materials availability

The cDNA for the S1 subunit of OZITX as described in the manuscript will be made available on reasonable request.
