# Supplementary material for: OZITX, a pertussis toxin-like protein for occluding inhibitory G protein signalling including Gαz
Source: Commun Biol. 2022 Mar 23;5:256. doi: 10.1038/s42003-022-03191-5 (PMC8943041; doi:10.1038/s42003-022-03191-5)
Supplement: Supplementary file 2 — Supplementary Information [file 42003_2022_3191_MOESM2_ESM.pdf]

Supplementary Information

OZITX, A pertussis toxin-like protein for occluding inhibitory G protein signalling including Gα_z

Alastair C. Keen*[1,2,3], Maria Hauge Pedersen*[4,5,6], Laura Lemel[2,3], Daniel J. Scott[7,8], Meritxell Canals[2,3], Dene R. Littler[9], Travis Beddoe[10], Yuki Ono[11], Lei Shi[12], Asuka Inoue[11], Jonathan A. Javitch^[4,5], J Robert Lane^[2,3]

[1]Drug Discovery Biology, Monash Institute of Pharmaceutical Sciences, Monash University, Parkville, Victoria, 3052, Australia.

[2] Division of Physiology, Pharmacology and Neuroscience, School of Life Sciences, Queen's Medical Centre, University of Nottingham, Nottingham, United Kingdom.

[3]Centre of Membrane Proteins and Receptors, University of Birmingham and University of Nottingham, Nottingham, United Kingdom.

[4]Departments of Psychiatry and Molecular Pharmacology and Therapeutics, Vagelos College of Physicians and Surgeons, Columbia University, New York, United States.

[5]Division of Molecular Therapeutics, New York State Psychiatric Institute, New York, United States.

[6]NNF Center for Basic Metabolic Research, Section for Metabolic Receptology, Faculty of Health and Medical Sciences, University of Copenhagen, Copenhagen, Denmark

[7]Department of Biochemistry and Molecular Biology, University of Melbourne, Parkville, Victoria, 3052, Australia

[8]The Florey Institute of Neuroscience and Mental Health, University of Melbourne, Parkville 3052, Victoria, 3052, Australia

[9]Infection and Immunity Program and Department of Biochemistry and Molecular Biology, Biomedicine Discovery Institute, Monash University, Clayton, Victoria, 3052, Australia

[10] Dept. of Animal, Plant and Soil Science and Centre for AgriBioscience, La Trobe University, Bundoora, Victoria, 3086, Australia

[11]Graduate School of Pharmaceutical Sciences, Tohoku University, Sendai, Miyagi 980-8578, Japan

[12]Computational Chemistry and Molecular Biophysics Section, National Institute on Drug Abuse - Intramural Research Program, National Institutes of Health, Baltimore, Maryland, United States

* these authors contributed equally

^These authors jointly supervised this work

Correspondence should be addressed to:

Rob Lane; rob.lane@nottingham.ac.uk,
Jonathan Javitch; Jonathan.Javitch@nyspi.columbia.edu

Supplementary Note 1

Codon optimized sequence of the active S1 subunit of OZITX (*Ec*PltAB)

ATGCTTAAAATGTTCATACTGTTTCTCATTAGCTTTTCATGGTATGCGAACGCTACCGAC
TTCGTATACAGGGTAGACTCCCGACCGCCTGAAGAAATTTTTCGAGACGGATTCCGCA
GCCACGGGTTTAATAGAAATCTGCAACAGCACCTGCGCGGGGATTCTTGCGCGGCCG
GCAGCCGGGACTCTGCGTTTATTGCCACCACTACCTCTCTGATTGAAACGTATAACATC
GCCAGGCAATATTACAGTAGCAGTGGTTTTCACGGACGCCTGTACCGCTATAGAATCC
GAGCCAATAATATCTTCTACCCAATCCAGCCAAGCGTTAACTATCTTACTCAGCGAGGT
ATAACTTTTTCTGGGTTCGAGCGCATCATGATGAGGGAACAAAACGAGATTGTAGCGGT
CGAGCACATACCGGGAGAGAATATCGTGGAGGCTGTTGAACTGACATACGACAGGTTT
AACAGCCAAGTCAGCGACGGACCCGGTACCACAAATGCGAGATACGTTCCTGGTAGCA
CCTTCGTTAACCCAGGCGTTATTCCACAACTCGTGGTGCCCACGGTATCCGTTCGAGAA
CGCATAAACGCATTCGGTTCACTCATTTCCGCATGTTTCGCCCTGAAAGGGGTGCGAA
GGGACGGTCTTAATAAGAGAGCCACATACTACGAGCCCGAATTTTACGACGCGCGAGG
CGTACTCAAGGAAATCATCAAGTGA

Supplementary Table 1. Primers for mutagenesis to create OZITX resistant Gα subunits.

Gα	C-terminal sequence	Primer Reverse with **XhoI** & **XbaI** digestion sites
$G\alpha_{i1}$	TDVIIKN**A**LKDCGLF	CTAG**CTCGAG**TTAAAAGAGACCACAATCTTTTAG**AGC**ATTTTTTATGATGACATC
$G\alpha_{i2}$	TDVIIKN**A**LKDCGLF	CTAG**CTCGAG**TTAAAAGAGACCACAATCTTTTAG**AGC**ATTTTTTATGATGACATC
$G\alpha_{i3}$	TDVIIKN**A**LKECGLY	CTAG**CTCGAG**TCAATAAAGTCCACATTCCTTTAA**GGC**GTTTTTAATGATGACATC
$G\alpha_{oa}$	TDIIIAN**A**LRGCGLY	CTA**TCTAGA**TCAGTACAAGCCGCAGCCCCGGAG**GGC**GTTGGCAATGATGATG
$G\alpha_{ob}$	TDVIIAK**A**LRGCGLY	CTA**TCTAGA**TCAGTAGAGTCCACAGCCCCGTAG**GGC**TTTGGCGATGATGACATCTG
$G\alpha_{z}$	TDVIIQN**A**LKYIGLC	CTAG**CTCGAG**TCAGCAAAGGCCAATGTACTTGAG**AGC**GTTCTGTATGATGAC

Supplementary Table 2. Primers to generate chimeric $G\alpha_{qi10}$ and $G\alpha_{qi13}$

	Target sequence	Forward primer	Reverse primer
$G\alpha_{qi10}$	AAAAATAATCTAAAAGATTGTG GTCTCTTT	GATTGTGGTCTCTTTTAACTCGAG TCTAGAGGG	TTTTAGATTATTTTTGAGGATGGT GTCCTTGAC
$G\alpha_{qi13}$	GTCATCATAAAAAATAATCTAA AAGATTGTGGTCTCTTT	AAAAGATTGTGGTCTCTTTTAACT CGAGTCTAGAGGGC	AGATTATTTTTTATGATGACGTCC TTGACGGCAGCAAA

a

```
OZITX_S1        -------------------MLKMFILFLISFSWYANATDFVYRVDSRPPEEIFRDGFRSH   41
PTX_S1          MRCTRAIRQTARTGWLTWLAILAVTAPVTSPAWADDPPATVYRYDSRPPEDVFQNGFTAW   60
                                    :  .   : * :*  :    *** ******::*::** :

OZITX_S1        GFNRNLQQHLRGDSCAAGSRDSAFIATTTSLIET--YN---------IARQYYSSSGFHG   90
PTX_S1          GNNDNVLDHLTGRSCQVGSSNSAFVSTSSSRRYTEVYLEHRMQEAVEAERAGRGTGHFIG   120
                * * *: :** * ** .** :***::*::*   *  *            *   .:. * *

OZITX_S1        RLYRYRIRANNIFYPIQPSV-NYLTQRGITFSG-FERIMMREQNEIVAVEHIPGENIVEA   148
PTX_S1          --YIYEVRADNNFYGAASSYFEYVDTYGDNAGRILAGALATYQSEYLAHRRIPPENIRRV   178
                  * *.:**:* **    *  :*:   * . .  :   :   *.* :* .:** *** ..

OZITX_S1        VELTYDRFNSQVSDGPGTTNARYVPGSTFVNPGVIPQLVVPTVSVRERINAFGSLISACF   208
PTX_S1          TRVYHNGITG-ETTTTEYSNARYVSQQTRANPNPYTSRR-SVASIVGTLVRMAPVIGACM   236
                ..: :: :..  :     :*****  .* .**.   .    ..*:   :  :. :*.**:

OZITX_S1        ALKGVRRD---------GLNKRATYYEPEFYDARGVLKEIIK    241
PTX_S1          ARQAESSEAMAAWSERAGEAMVLVYYESIAYSF---------    269
                * :.   :         *     .***   *.
```

b

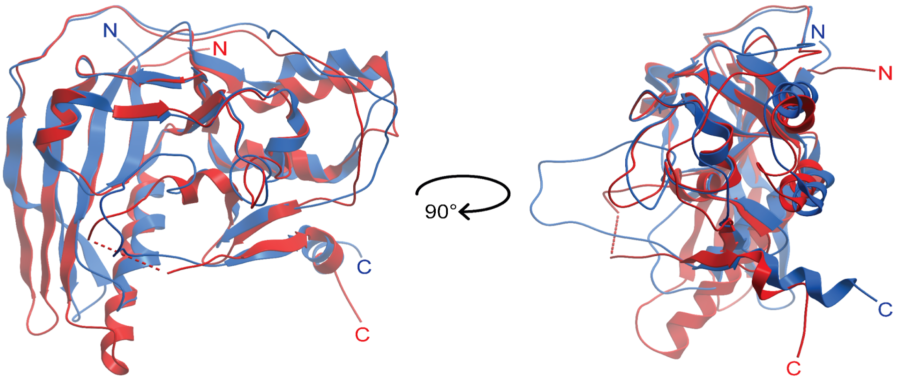

Supplementary Figure 1: Structural comparison of OZITX-S1 and PTX-S1. (**a**) Amino acid sequence alignment of OZITX-S1 and PTX-S1 active subunits. Sequences were aligned with Clustal Omega version 1.2.4. '*' represents a completely conserved residue. ':' represents a conserved residue (>0.5 in the Gonnet PAM 250 matrix). '.' represents a weakly conserved residue (≤0.5 and >0 in the Gonnet PAM 250 matrix). (**b**) Structural superimposition of OZITX-S1 and PTX-S1 active subunits. OZITX-S1 is depicted in blue cartoon (PDB code: 4Z9C). PTX-S1 is depicted in red cartoon (PDB code: 1PRT). The toxins are presented in the oxidised state; the state whereby the A subunit is bound to the B subunits. The B subunits have been removed for clarity.

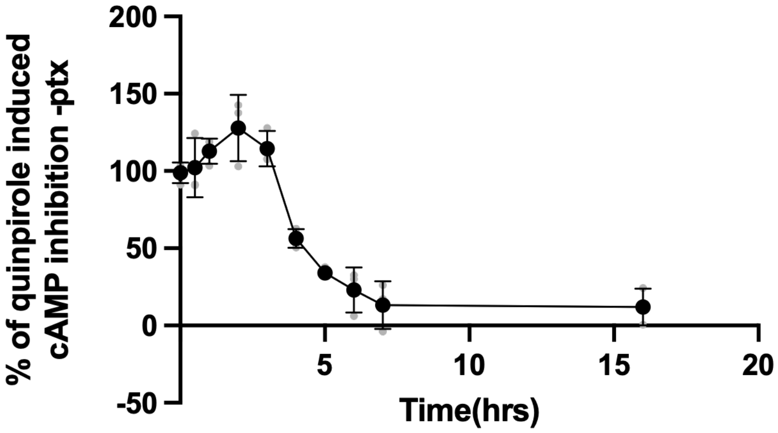

Supplementary Figure 2: Timecourse for PTX inhibition of quinpirole induced cAMP inhibition. (a) HEK293 cells transfected with DNA encoding D_2R and CAMYEL were incubated with vehicle or PTX at different timepoints prior to running the cAMP assay. Cells were pre-stimulated with 10 *μ*M forskolin for 10 minutes before stimulation with either vehicle control or 10 μM quinpirole. Data are normalized to quinpirole induced cAMP inhibition without PTX stimulation and is shown as the mean ± SD from 3 separate experiments conducted in triplicates.

a

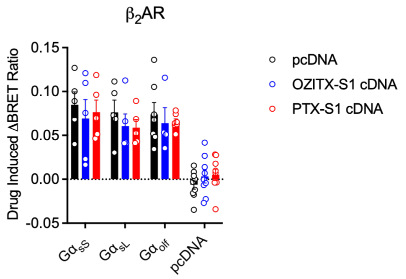

b

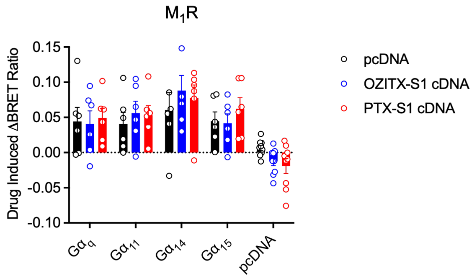

c

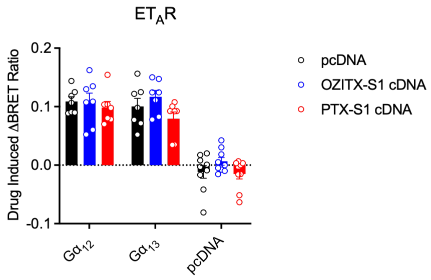

Supplementary Figure 3: Gα_s, Gα_q and Gα_{12} subfamily activation upon transfection of OZITX active subunit. (**a**) β_2-adrenergic receptor (β_2AR) activation of Gα_{sS}, Gα_{sL} and Gα_{olf}, n=4. (**b**) M_1 muscarinic acetylcholine receptor (M_1R) activation of Gα_q, Gα_{11}, Gα_{14} and Gα_{15}, n=6. (**c**) endothelin A receptor (ET$_A$R) activation of Gα_{12} and Gα_{13}, n=7, where pcDNA indicates a condition without overexpressed G protein. HEK 293T cells were transfected with cDNA encoding the particular GPCR, the Gα, the G protein activation sensors and either pcDNA3.1+ control (black), OZITX-S1 cDNA (blue) or PTX-S1 cDNA (red) as described in the methods. 48 hours after transfection the BRET assay was performed, stimulation of the cells was carried out by adding the agonists isoproterenol (β_2AR) / acetylcholine (M_1R) / endothelin 1 (ET$_A$R) for 5 minutes followed by BRET detection. The data are represented as the mean ± SEM drug induced increase in BRET ratio from the vehicle control.

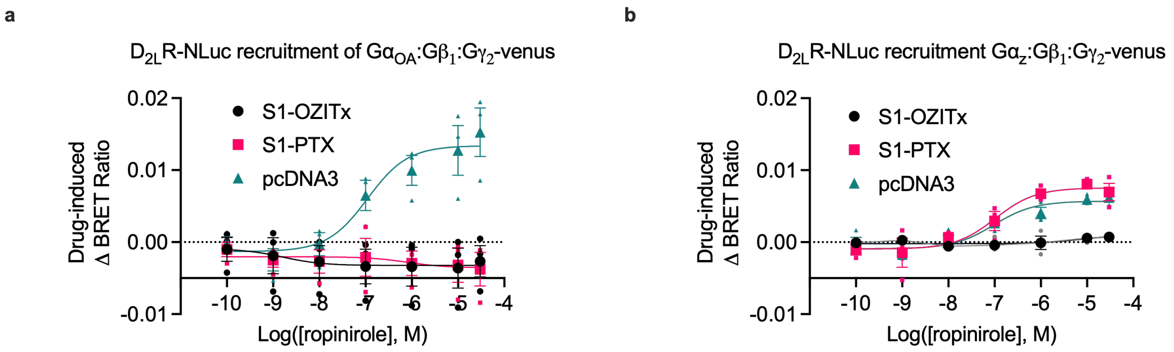

Supplementary Figure 4: Gα_{oA}:β_1:γ_2-venus (a) or Gα_z:β_1:γ_2-venus (b) recruitment to the D_{2L}R fused to nanoluciferase (D_{2L}R-NLuc) at its C-terminus. HEK293 cells were transiently transfected with plasmids encoding D_{2L}R-NLuc, Gα_{oA} or Gα_z, Gβ_1 and Gγ_2-venus and either the catalytic subunit of PTX (S1-PTX – pink squares), OZITx (S1-OZITx, black circles) or pcDNA3 as a control (teal triangles). The ability of increasing concentrations of the D_2R agonist ropinirole to cause recruitment of the Gα_{oA}:β:γ-venus or Gα_z:β:γ-venus heterotrimer to D_{2L}-Nluc was measured as an increase in BRET ratio. The data are represented as the mean ± SEM drug induced increase in BRET ratio from the vehicle control. Each data point is the mean from 3 individual experiments performed in triplicate.

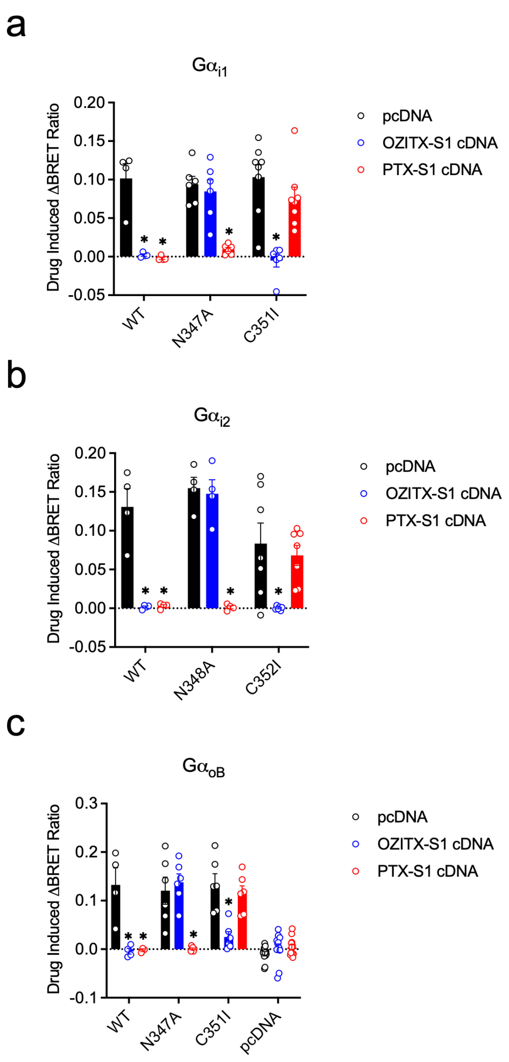

Supplementary Figure 5: OZITX Resistant mutants of $G\alpha_{i1}$, $G\alpha_{i2}$ and $G\alpha_{oB}$ can be engineered. (**a**) Activation of $G\alpha_{i1}$-WT, $G\alpha_{i1}$-N347A and $G\alpha_{i1}$-C351I, n = 4. Data are shown as the mean ± SEM. * represents the response is significantly different from the respective pcDNA (non-toxin transfected) control response (black bar) using a one-way ANOVA with Dunnett's multiple comparisons test, $P < 0.05$. (WT: pcDNA vs OZITX – P = 0.0002, pcDNA vs PTX – P = 0.0002. N347A: pcDNA vs PTX $P < 0.0001$. C351I pcDNA vs OZITX – $P < 0.0001$). (**b**) Activation of $G\alpha_{i2}$-WT, $G\alpha_{i2}$-N348A and $G\alpha_{i2}$-C352I, n = 9. (WT: pcDNA vs OZITX – P = 0.0003, pcDNA vs PTX – P = 0.0002. N347A: pcDNA vs PTX $P < 0.0001$. C351I pcDNA vs OZITX – P = 0.009). (**c**) Activation of $G\alpha_{oB}$-WT, $G\alpha_{oB}$-N347A and $G\alpha_{oB}$-C352I, n = 12. (WT: pcDNA vs OZITX – P = 0.004, pcDNA vs PTX – P = 0.007. N347A: pcDNA vs PTX P = 0.0009.

C351I pcDNA vs OZITX – P = 0.0008). G protein activation was performed in the presence of either transfected pcDNA3.1+ control (black), OZITX-S1 cDNA (blue) and PTX-S1 cDNA (red). HEK 293 cells were transfected with the Gα mutant subunit of interest or pcDNA control, G protein activation sensors, the D_2R and either a pcDNA3.1+ control, OZITX-S1 cDNA or PTX-S1 cDNA. Cells were then stimulated with quinpirole and the drug induced increase in BRET ratio baseline subtracted from the vehicle wells is represented. Data are shown as the mean ± SEM. * represents the response is significantly different from the respective pcDNA (non-toxin transfected) control response (black bar) using a one-way ANOVA with Dunnett's multiple comparisons test.

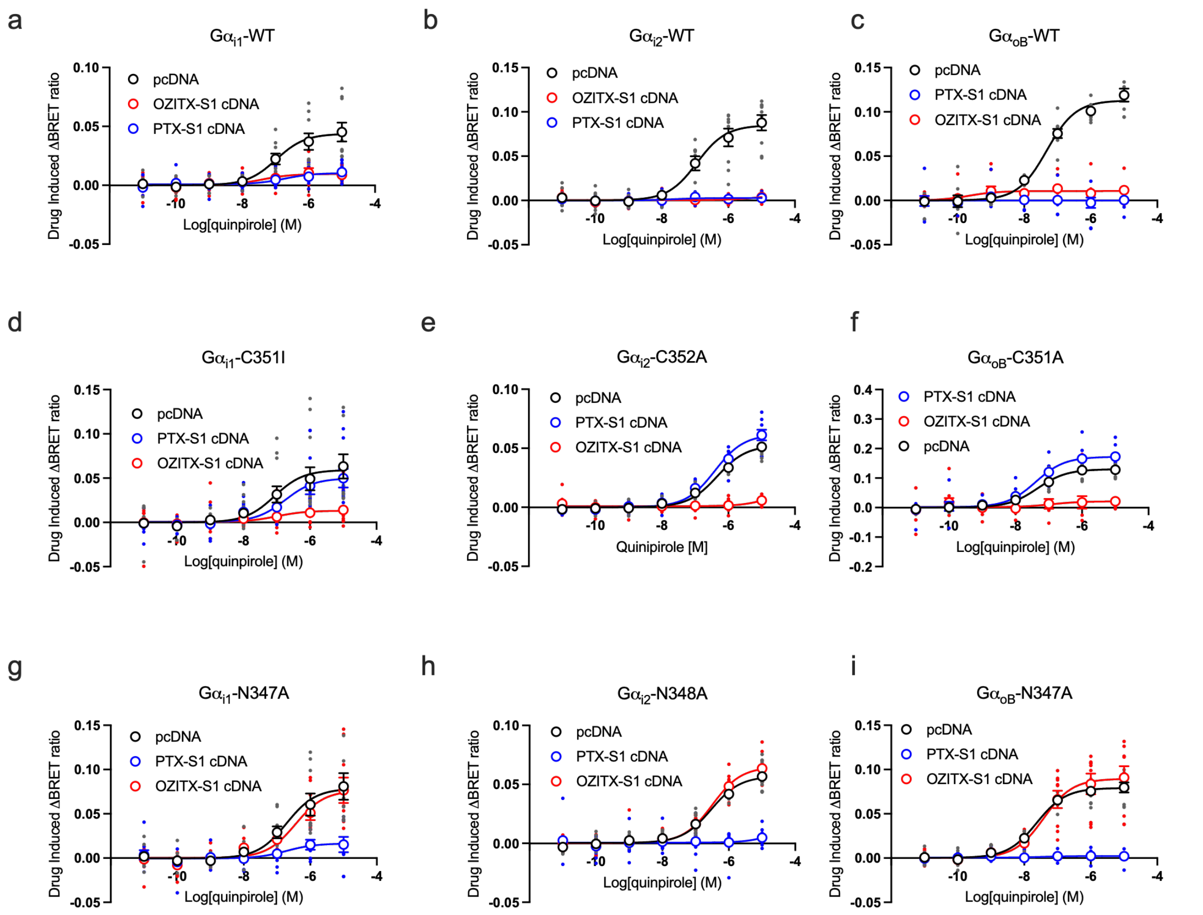

Supplementary Figure 6: OZITX Resistant mutants of $G\alpha_{i1}$, $G\alpha_{i2}$ and $G\alpha_{oB}$ can be engineered. (a) $G\alpha_{i1}$-WT activation, n = 9. (b) $G\alpha_{i2}$-WT activation, n = 9. (c) $G\alpha_{oB}$-WT activation, n = 9. (d) $G\alpha_{i1}$-C351A activation, n = 12. (e) $G\alpha_{i2}$-C352A activation, n = 12. (f) $G\alpha_{oB}$-C351A activation, n = 9. (g) $G\alpha_{i1}$-N347A activation, n = 9. (h) $G\alpha_{i2}$-N348A activation, n = 12, (i) $G\alpha_{oB}$N347A. The G protein activation assay was performed on WT, Asn347Ala/Asn348Ala (putative OZITX site) and Cys351Ile/Cys352Ile (PTX insensitive) mutants. Cells were transfected with the D_2R, the particular Gα mutant, the G protein activation sensors and either a pcDNA3.1+ control (black open circles), OZITX-S1 cDNA (blue open circles) or PTX-S1 cDNA (red open circles). Cells were then stimulated with increasing concentrations of quinpirole before BRET detection. Data represent the mean drug induced increase in BRET ratio from vehicle ± SEM.

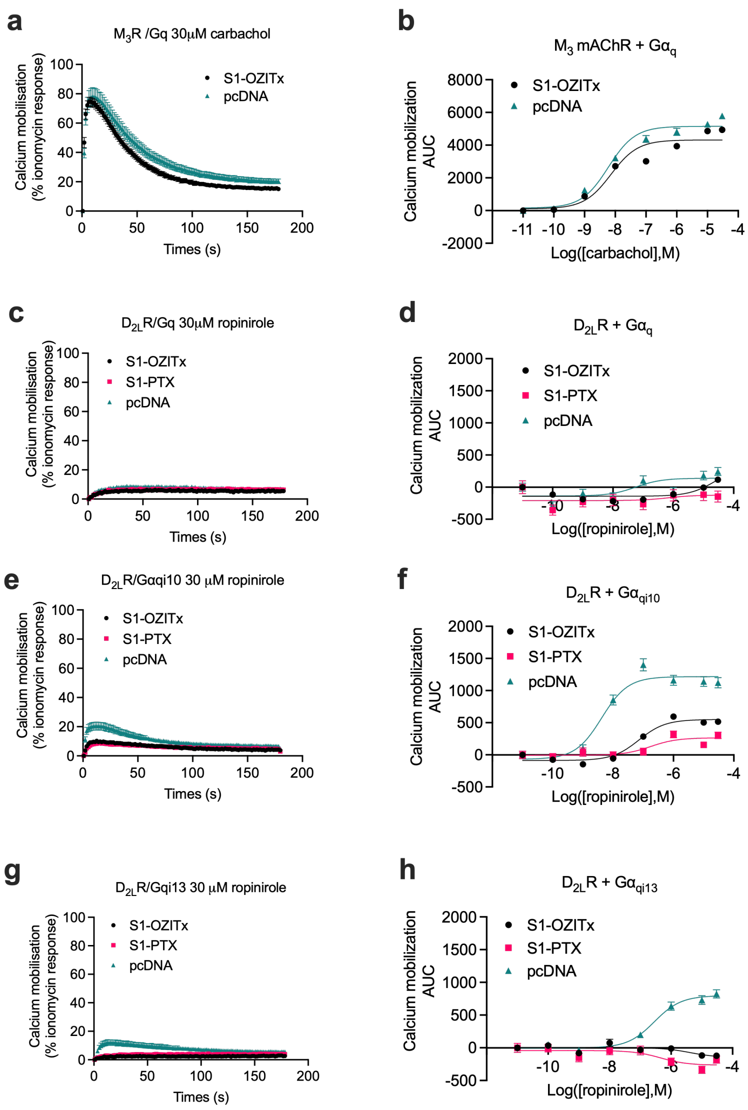

Supplementary Figure 7: The C-terminal 10 amino acids of Gα$_i$ are sufficient to confer OZITX selectivity. HEK293 cells were transfected with plasmids encoding the muscarinic M$_3$ acetylcholine receptor (M$_3$ mAChR) and Gα$_q$ (a & b, n = 15) or the D$_{2L}$R and Gα$_q$ (c & d, n = 15) or chimeras Gα$_{qi10}$ (e & f), in which last 10 amino acids of Gα$_q$ were replaced with those of Gα$_{i3,}$ n = 15) and Gα$_{qi13}$ (g & h, in which last 13 amino acids of Gα$_q$ were replaced with those of Gα$_i$ and the catalytic subunit of PTX or OZITX, n = 12). The Ca^{2+} mobilisation response to 30 µM carbachol (A) or ropinirole (c, e, g) was measured over 180 seconds. The area under the curve (AUC) of Ca^{2+} mobilisation responses over 180 seconds to increasing concentrations of carbachol (b) or ropinirole (d,f,h) were measured.

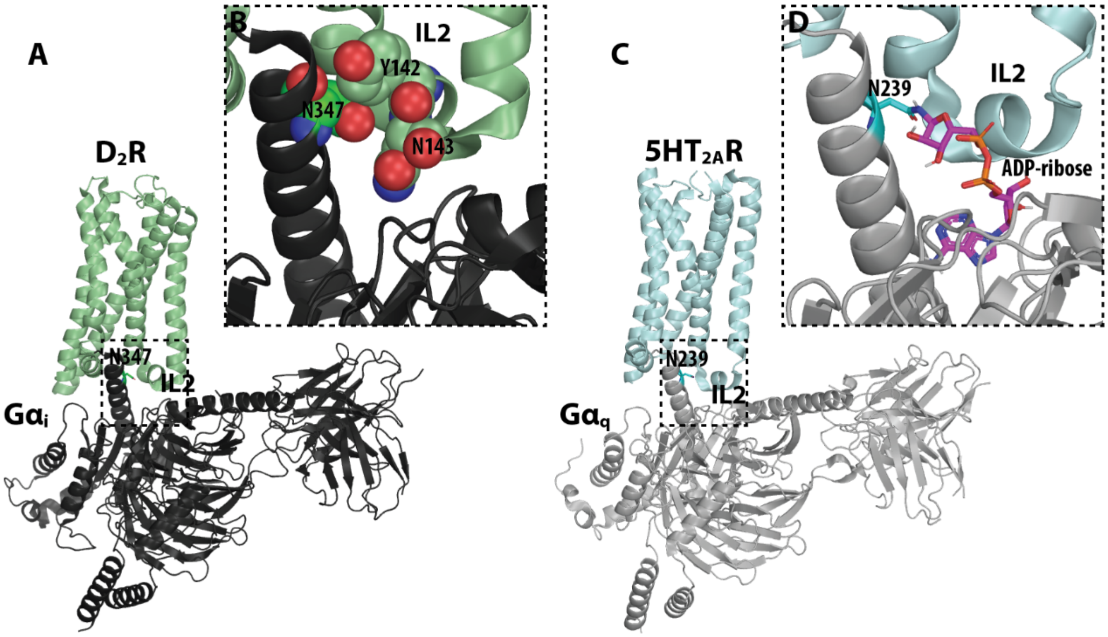

Supplementary Figure 8. The $5HT_{2A}R$-$G\alpha_q$ interface may accommodate ADP-ribosylation of the C terminal asparagine of $G\alpha_q$. In the cryo-EM structure of D_2R-$G\alpha_i$ complex (PDB 7JVR)[49], Asn[347], eight residues from the C-terminus of $G\alpha_i$, is in close proximity to intracellular loop 2 (IL2) of D_2R (a), and is tightly packed with Tyr[142] and Asn[143] of IL2 (b), which prevented ADP-ribosylation of the residue in our covalent docking study. In contrast, in the $5HT_{2A}R$-$G\alpha_q$ complex (PDB 6WHA)[48], the aligned Asn[239] in the engineered $G\alpha_q$ is far from IL2 of $5HT_{2A}R$ and faces a more open space (c), which allows easy in silico ADP-ribosylation of Asn[239]. Multiple possible poses of the covalently attached ADP-ribose moiety can be generated in our docking study, a representative pose is shown in panel (d). The covalent docking was carried out with the covDock module of Schrodinger suite (version 2021-1), assuming a SN2 nucleophilic substitution reaction, which results in the α-orientation of the attached ADP-ribose moiety on the asparagine[52]. It should be noted that the $5HT_{2A}R$ structure is in complex with an engineered $G\alpha_q$ protein in which the N-terminal 35 residues are replaced by the corresponding $G\alpha_{i2}$ residues[48], which are only minimally involved in the receptor-G protein interface.
